# Associations Between Adiposity Measures and Depression and Well-Being Scores: A Cross-Sectional Analysis of Middle- to Older-Aged Adults

**DOI:** 10.1192/bjo.2024.196

**Published:** 2024-08-01

**Authors:** Caoimhe Lonergan, Seán Millar, Zubair Kabir

**Affiliations:** 1 Cork University Hospital, Cork, Ireland; 2 University College Cork, Cork, Ireland

## Abstract

**Aims:**

Obesity and mental health are significant global health concerns. Evidence has linked increased adiposity with depression and well-being; however, there is limited documented evidence in Ireland. Research also suggests that lifestyle factors and disease conditions are related to mental health. These may modulate relationships between adiposity and depression and well-being. The aim of this study was to examine associations between mental health scores and adiposity defined using body mass index (BMI) and waist-height ratio, and subsequently determine whether significant relationships persist following adjustment for lifestyle factors and common disease conditions.

**Methods:**

This was a cross-sectional study of 1,821 men and women aged 46–73 years, randomly selected from a large primary care centre. Depression and well-being were assessed using the 20-item Centre for Epidemiologic Studies Depression Scale (CES-D) and the World Health Organization-Five (WHO-5) Well-Being Index. Linear regression analyses were performed to examine relationships between mental health scores (dependent variable) and adiposity defined using BMI and waist-height ratio (independent variable), while adjusting for demographic characteristics, lifestyle factors and disease conditions. These demographic, lifestyle and disease factors included gender, age, education, smoking status, alcohol intake, physical activity levels, dietary quality, type 2 diabetes, cardiovascular disease and cancer.

**Results:**

BMI and waist-height ratio had a significant positive association with depression scores and a significant inverse association with well-being scores in males and females. These associations were maintained following adjustment for demographic variables and lifestyle factors. In final models where disease conditions were adjusted for, BMI (β = 0.743, p <0.001) and waist-height ratio (β = 0.719, p <0.001) associations with the CES-D score remained significant. In stratified analyses, relationships between measures of adiposity and depression were found to be stronger in females (BMI: β = 0.806, p = 0.007; waist-height ratio: β = 0.768, p = 0.01) than males (BMI: β = 0.573, p = 0.049; waist-height ratio: β = 0.593, p = 0.044) but no effect modification was identified.

**Conclusion:**

This study demonstrates a significant association between increased adiposity and poorer mental health in a middle- to older-aged population, which is in agreement with previous evidence. In addition, these findings suggest that the positive relationship between adiposity and depression is independent of lifestyle factors and disease conditions and is stronger in females. Targeted interventions for reducing depression should include better weight management population-level measures, particularly in the female population.

